# Characterizing TV viewing habits in companion dogs

**DOI:** 10.1038/s41598-025-06580-y

**Published:** 2025-07-17

**Authors:** Lane I. Montgomery, Sarah Krichbaum, Jeffrey S. Katz

**Affiliations:** 1 Department of Psychological Sciences, Auburn University, 226 Thach Hall, Auburn, AL 36849 USA; 2 Canine Performance Sciences, College of Veterinary Medicine, Auburn University, Auburn, AL 36849 USA

**Keywords:** Television, Dogs, Temperament, Welfare, Psychology, Sensory processing, Animal behaviour

## Abstract

Televisions provide a unique perceptual experience to dogs, which can result in added enrichment (e.g., mental stimulation) or stressors (e.g., reactivity towards images) in a dog’s day to day life. However, little research has sought to quantify and identify the mechanisms responsible for television viewing behaviors in dogs. A novel Dog Television Viewing Scale (DTVS) was distributed to companion dog owners. Questions were related to the types of stimuli the dog behaviorally reacted to (animals, inanimate objects) and the sensory domains through which these stimuli were perceived (vision, audition). A principal component analysis was conducted on the DTVS items using 453 responses. Generalized linear models were conducted to examine the impact of demographic and temperament factors on component scores. Notably, DTVS scores were best explained by components related to the types of stimuli (animals, inanimate objects) present on the television and the types of behavior the dog exhibited (following behaviors, stationary behaviors). Furthermore, measures of certain temperament traits assessed by dog owners, namely excitement and negative reactivity, were predictive of component scores. These results suggest that television viewing habits may be predicted by aspects of a dog’s personality and that dogs may represent objects in the 2D television format similarly to the 3D environment. These results inform toward the perceptual experience of companion dogs and potential dog welfare interventions (e.g., shelter dog enrichment). In practice, engagement with television could provide dogs with an enriching, meaningful experience.

**Supplementary Information:**

The online version contains supplementary material available at 10.1038/s41598-025-06580-y.

## Introduction

As companion animals, dogs are exposed to many forms of artificial environmental stimuli that would not be encountered in the wild. Of note, televisions provide 2D images of naturalistic stimuli that on both an auditory and visual level can be characterized as lifelike. Because companion dogs are regularly exposed to televisions, it is pertinent to understand how dogs behaviorally respond to and interpret these types of stimuli from a welfare perspective. With media-related technology more accessible than ever, the frequency with which dogs are exposed to different forms of media is increasing. Not only is it more common for dogs to be exposed to media, but television quality is increasing in a way that makes the stimuli from these devices fit more seamlessly into the surrounding environment. Furthermore, there has been a rise in dog-specific television programming (i.e., programming designed to be ecologically relevant to dogs) in recent years. Not only do many owners play television programs for their dogs, but brands have developed that market television programs specifically designed with dogs in mind (e.g., DOGTV®). Informal observations suggest that dogs attend to the television and behaviorally respond to television stimuli; however, there have been few peer-reviewed studies that support these claims^1,2^.

Thus far, no peer-reviewed study has provided population-level information on how many dogs attend to televisions. However, Blackwell et al.^3^ examined behaviors suggestive of fear in dogs, with television-related responses being behaviors of interest. In the sample of fearful dogs, only around 15% of dogs would respond to television. Another study conducted by Wells^4^ examined problem behaviors such as barking at televisions. Interesting, only one third of dogs with extreme barking behaviors would bark at the television. While these studies suggest low rates of television attendance in dogs, their goal was not to characterize television viewing habits, and the samples were comprised of subsets of dogs exhibiting problem behaviors. However, some organizations have conducted surveys designed to examine television viewing rates. The Center for Canine Behavior Studies, in a 2021 survey, found that 50% of dogs in their 708-dog sample would react to televisions. Similarly, according to the National Broadcasting Company (NBC), a study conducted by the company IAMS™ and the American Kennel Club (AKC) purportedly found that around 50% of dogs displayed interest in televisions. However, these studies were not peer-reviewed, being performed for the purposes of the organizations. Thus, strong conclusions upon television viewing rates cannot be gleaned from these studies alone.

Likewise, there have been a limited number of studies to examine the types of television stimuli with which dogs will engage. In a study conducted by Pongrácz et al.^5^, dogs would equally obey commands when presented via a present human (3D) or through a video recording of a human (2D), indicating that dogs are able to interpret information through a 2D modality. Another study found that when dogs were given access to televisions with different types of media (i.e., human stimuli, conspecific stimuli, heterospecific stimuli), dogs would engage more with “relevant” (e.g., dogs and humans) stimuli^1^. Though these authors suggest that even when dogs are able to purposefully alter television activity, they seldom engage with the media^2^.

Current research examining dog television watching remains limited, as studies often are hindered by small samples sizes^2,5^ or the examination of television viewing with specific behavioral issues in mind^3,4^. While it is known that dogs’ perception of television stimuli is different than that of humans^6^, it is unclear how or whether dogs use and process that information. Furthermore, it is not known what factors influence a dog’s likelihood of engaging with television stimuli. Understanding of such behaviors would provide a more comprehensive picture of dogs’ perceptual experiences, with practical applications for visual presentations in experimental settings. Further, understanding television viewing behaviors could guide usage of television programming as a form of companion or shelter dog enrichment, clarifying the ideal stimulus categories for such activities.

To our knowledge, there has been no systematic, peer-reviewed investigation of television viewing habits in dogs. In addition, the impacts of factors such as age, breed group, sex, and temperament on television viewing behaviors have not been examined. Beyond species-level similarities in perception, individual characteristics related to the dog (e.g., breed background, attentional capacities) may influence their attendance to television (see specific hypotheses below). To begin to address these issues, we distributed a survey containing temperament scales (Positive and Negative Activation Scale (PANAS)^7^ and Dog Impulsivity Assessment Scale (DIAS)^8^), a novel Dog Television Viewing Scale (DTVS), and demographic questions to a population of companion dog owners. A principal component analysis (PCA) was conducted on the DTVS in order to identify which characteristics of television stimuli represent underlying differences in television viewing. Then, the influence of individual characteristics upon these components was examined.

We hypothesized that older dogs would attend to television stimuli less than younger dogs due to greater experience with television stimuli. We hypothesized that PCA component loadings would relate to the modality through which the stimulus was perceived (i.e., vision, audition) and how ecologically relevant the stimulus is to the dog (e.g., dogs and other household animals loading together). In addition, we expected that dogs with greater levels of negative reactivity would be more likely to respond to television stimuli, in line with previous research finding (albeit low rates of) negative behavioral responses to television stimuli^3,4^. Furthermore, we hypothesized that dogs with higher impulsivity levels would respond more to television stimuli due to lower levels of behavioral regulation. Breed group was included as an exploratory analysis, based upon the fact that different breeds have different historical functions (e.g., hounds were bred to rely upon olfactory discrimination while herding dogs were bred to rely upon visual discrimination), which may influence television viewing behaviors. Lastly, we did not expect to see any differences in viewing behaviors as a result of sex, given that prior studies have not reported a significant sex effect.

## Methods

### Subjects

Companion dog owners were recruited for participation in this study via social media posts. Dog owners completed the study anonymously online via a Qualtrics® survey link or QR code on social media from February 2024 to March 2024. Location information was not collected, but the survey was not restricted to residents of the United States. Informed consent was obtained at the beginning of the survey. In total, responses for 650 dogs were collected. After incomplete and duplicate responses were removed, 513 responses remained. Once dogs with “no” responses to the “does your dog watch television” question were removed, 453 responses remained (Sex: M = 233; Age: *M* = 4.27, SEM = 0.15). Hence, 88.3% of dogs in the original sample watched TV. While viewing behaviors of dogs who generally do not engage with the television are valuable, self-selection of owners with dogs who watch television did occur and therefore the small number of no responses (n = 32) were excluded. The age range of the final sample varied from four months to 16 years, with a mean age of 4.27. The amount of time the owner had possessed the dog ranged from two months to 16 years. Three hundred dogs represented breeds that were recognized by the AKC, while 153 dogs were mixed breeds or breeds that are not formal members of AKC groupings. All seven AKC breed groupings were represented in the sample (Herding = 98; Hound = 15; Non-sporting = 34; Sporting = 81; Terrier = 26; Toy = 16; Working = 30). All combinations of sex and neuter status were represented in the sample (intact females = 61; spayed females = 159; intact males = 80; neutered males = 153). All methods were approved by Auburn University’s Institutional Review Board (Protocol # 23-666 EX 2402) and conducted in accordance with all relevant guidelines and regulations.

### Survey measures

The distributed survey included demographic questions (i.e., age, sex, breed, neuter status), questions concerning the dog’s general TV viewing habits (see Supplementary Materials Table S1), the Positive and Negative Activation Scale (PANAS), the Dog Impulsivity Assessment Scale (DIAS), and the novel Dog Television Viewing Scale (DTVS). Questions concerning the dog’s general TV habits included questions concerning whether or not the owner tried to teach the dog to watch TV (binary variable: TV teach), the average number of hours per week the owner’s TV is turned on (continuous variable: TV exposure), and the average number of seconds (converted from the owner’s chosen unit of time) that the dog will attend to the TV (continuous variable: attend length). The response percentages for the additional TV questions (Table S1) and the DTVS (Table S2) are located in the Supplementary Materials.

The PANAS consists of 21-items on a 5-point Likert type scale (strongly disagree to strongly agree) and is made up of 5 subscales (Negative Activation and Overall Positive Activation, the latter of which is further divided into Energy and Interest, Persistence, and Excitability), with higher subscale scores representing higher levels of the trait^7^. Negative Activation refers to fearful reactions to stimuli and changing environments; Overall Positive Activation and its components refer to dogs’ responses to rewarding stimuli^9^.

The DIAS consists of 18-items on a 5-point Likert type scale (strongly disagree to strongly agree) and is made up of 4 subscales (Behavioral Regulation, Response to Novelty, Responsiveness, and Overall score), with higher subscale scores representing higher levels of the trait, except for Behavioral Regulation in which higher scores represent lower levels of the trait (i.e., higher impulsivity)^8^.

The DTVS (see Table 1) consisted of 16 questions concerning dogs’ interactions with televisions over two sensory domains: vision and audition. Questions were rated on a 5-point Likert scale ranging from “Never” to “Always.” Participants rated the frequency of specified behaviors toward the television for different types of stimuli. Different types of stimuli were included to allow for examination of how responses may vary based upon the ecological relevance of different items. For stimuli that the dog had not encountered through the television, a “Not Observed” selection choice was included. Items were rated from one to five, with one indicating a dog never displaying a behavioral response to a stimulus category and five indicating a dog always displaying a behavioral response to a stimulus category. For responses marked as “Not Observed,” these responses were rated as a zero. Inserting this dummy value allowed incomplete responses to be included while not skewing the statistical analysis with use of a meaningful number.

**Table 1:** The dog television viewing scale (DTVS).

	Never	Rarely	Sometimes	Often	Always	Not observed
For the following visual TV items, please state how often your dog follows those types of objects off-screen. Following includes (but is not limited to) behaviors such as looking behind the TV for an object and walking alongside an object as it leaves the screen						
Dogs						
Non-dog household pets (e.g., cats)						
Non-household animals (e.g., horses, birds)						
Humans						
Inanimate objects (e.g., cars)						
For the following visual TV items, please state how often your dog responds to those types of objects. Responding includes (but is not limited to) behaviors such as pawing at the TV, tail wagging, ear movements, and growling						
Dogs						
Non-dog household pets (e.g., cats)						
Non-household animals (e.g., horses, birds)						
Humans						
Inanimate objects (e.g., cars)						
For the following auditory TV items, please state how often your dog responds to those types of noises. Responding includes (but is not limited to) behaviors such as tail wagging, ear movements, barking, and whining						
Dog noises (e.g., barking, howling)						
Non-dog household animal noises (e.g., meowing)						
Non-household animal noises (e.g., neighing, mooing)						
Human noises (e.g., talking, yelling)						
Inanimate object noises (e.g., car horn, doorbell)						
Weather noises (e.g., thunder, rain)						

For the visual questions, owners first rated the frequency with which dogs followed an object off the television screen for five types of stimuli: dogs, non-dog household pets, non-household animals, humans, and inanimate objects. These questions were included to capture visual interactions with the television that are suggestive of an expectation that the object on the television is a part of the 3D visual environment. For the second portion of the visual component, owners rated the frequency with which dogs behaviorally responded to objects on the television (e.g., barking, change in body posture, tail wagging) for five types of stimuli: dogs, non-dog household pets, non-household animals, humans, and inanimate objects. These questions were included to capture visual interactions with the television that demonstrate engagement with the 2D stimuli, but that are not necessarily indicative of an expectation that that object exists in the 3D environment. Stimulus categories were purposefully kept broad, as it was expected that further parsing of categories would result in a larger number of “Not Observed” responses. However, the inclusion of various types of animal classes (i.e., dog, non-dog household pets, non-household animals, humans) was conducted so that ecologically significant differences in species (e.g., a dog is probably more familiar with a cat than a cow) were still captured.

For the auditory questions of the scale, owners rated the frequency with which dogs behaviorally responded (e.g., barking, change in body posture, tail wagging) to six types of auditory stimulus categories: dog noises, non-dog household pet noises, non-household animal noises, human noises, inanimate object noises, and weather noises. These questions were included to capture behavioral uses of auditory stimuli from the television, which may or may not be indicative of an expectation that that sound originates from the 3D environment.

### Statistical analyses

#### Principal component analysis

**Fig. 1:**
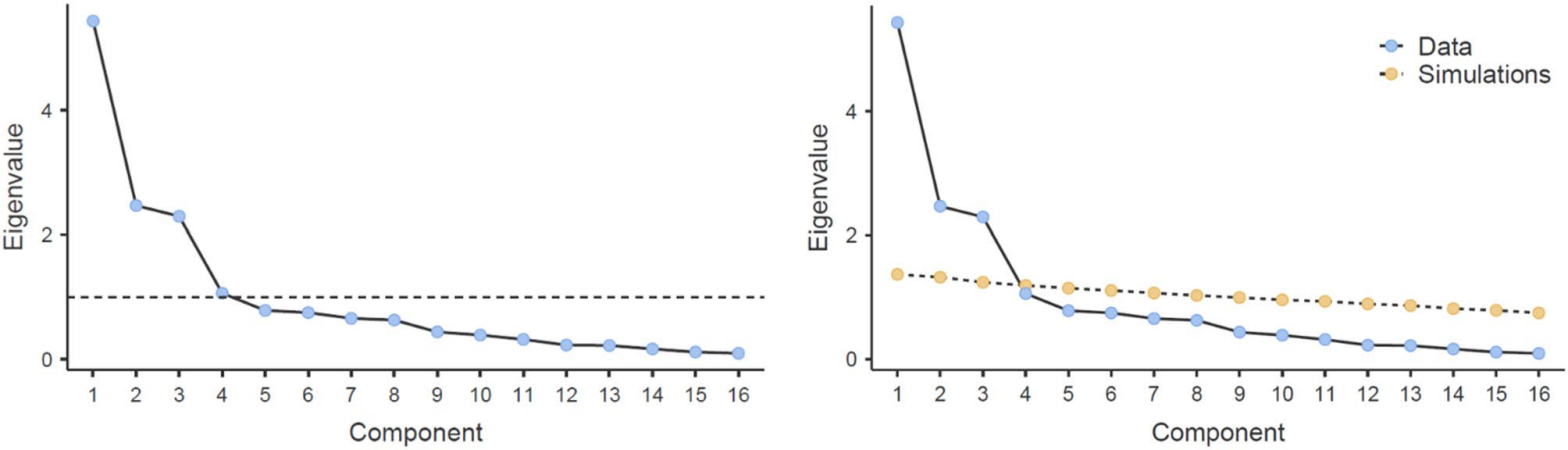
The scree plots for the PCA. The left scree plot represents component selection based upon eigenvalues greater than one. The right scree plot represents component selection based upon parallel analysis.

All statistical analyses were conducted in Jamovi (version 2.6.11) and graphical creation was conducted in Jamovi (version 2.6.11) and R (version 4.3.1). A PCA was conducted upon the 16 questions of the DTVS with a varimax rotation and factor loadings below 0.4 suppressed, in line with previous survey validation studies^8^. Sampling adequacy of PCA was confirmed by Bartlett’s sphericity tests and Kaiser–Meyer–Olkin (KMO) measures. The final components were determined based upon parallel analysis and loadings of 0.4 or higher. While traditionally PCA criterion is based upon eigenvalues greater than one, parallel analysis was chosen due to its reliance on statistical theory and incorporation of sampling error^10^. Parallel analysis determines the appropriate number of factors via simulations that reflect the characteristics of the dataset, therefore limiting the potential of chance influencing the number of factors and taking into consideration the limitations of the dataset^10^. Conducting the PCA with the eigenvalue criteria resulted in four components, while conducting the PCA with parallel analysis resulted in three components. The loadings for the third and fourth components based upon eigenvalue differed from the loadings for the third component based upon parallel analysis, with the first two factor loadings remaining the same. Given the outlined statistical reasoning and the biological interpretations of the components, parallel analysis was used for the PCA. The scree plots based upon both the PCA parallel analysis and eigenvalue can be seen in Fig. 1. Per previous studies using a missing data cutoff^8^, survey scale items for which more than 5% (n = 22) of the sample responded, “Not Observed” were removed from analysis, however, this did not apply to any of the survey items. The only items for which such responses occurred more than once were the question concerning non-household animal noises (0.09% of respondents) and the question concerning weather noises (1.3% of respondents). No respondent replied “Not Observed” for more than one question. PCA component scores were used as subscale measures of the DTVS for all further analyses.

#### Generalized linear models

Generalized linear models (GLMs) with a gaussian family and identity link function were conducted with the dependent variables of DTVS component scores. First, a prior experience model containing the independent variables of TV teach and TV exposure was conducted to determine whether these prior experiences influence television-related behaviors. A model containing the independent variable of attend length (*M* = 848 s, SEM = 69.1) was then conducted for the component scores to provide validity to the survey measures, as dogs who attend to television stimuli for a longer amount of time should have higher PCA scores.

In order to identify the impact of individual characteristics upon television-related behaviors, a model was conducted to examine the impacts of age, sex, breed group, and neuter status upon component scores. One-way Welch’s ANOVAs were conducted for the variables of breed grouping and neuter status for each of the component scores to determine the necessity of including these variables in the model, with items with *p*s > 0.05 excluded. Given significant correlations between age, the PANAS subscale scores, and the DIAS subscale scores, PANAS subscale items and DIAS subscale items were separated into individual models. For any model containing multiple variables, interactions were included and removed stepwise.

## Results

### Principal component analysis

The 16 variables loaded onto three components that met the outlined criteria (Bartlett’s sphericity *x*^2^ = 4601, *p* < 0.001, KMO = 0.795). The first component accounted for 24.5% of the total variance and consisted of questions related to non-human animal (both conspecific and heterospecific) stimuli, therefore it was labelled “DTVS Animal.” The second component accounted for 21.4% of the total variance and consisted of questions related to following behaviors, therefore it was labelled “DTVS Follow.” Lastly, the third component accounted for 17.8% of the total variance and consisted of questions related to inanimate objects and humans, therefore it was labelled “DTVS Non-Animal.” The three components explained 63.7% of the total variance. None of the components significantly correlated with each other. Within the sample, DTVS Animal scores were normally distributed, indicating that the majority of dogs displayed a moderate level of responding to animal stimuli. The DTVS Follow scores displayed a flat distribution, indicating that the level of responding was evenly spread across the sample. Lastly, the DTVS Non-Animal scores were right-skewed, indicating that most dogs exhibited low levels of responding to these types of stimuli. The component loadings (Table S4) and the distribution of component scores (Fig. S1) are displayed in Supplementary Materials.

### Generalized linear models

#### DTVS animal

For the prior experience model, there were no significant effects, suggesting that prior environmental experiences did not influence animal viewing behaviors. Attend length had a significant effect on DTVS Animal scores, such that dogs with higher attendance lengths had higher DTVS Animal scores on average (*z* = 2.57, *p* = 0.01). Breed grouping and neuter status did not have significant effects on DTVS Animal scores, and thus were excluded from the individual characteristics model. Neither age nor sex had a significant effect on component scores. Lastly, none of the PANAS or DIAS subscale items had a significant effect on DTVS Animal scores.

#### DTVS follow

**Fig. 2:**
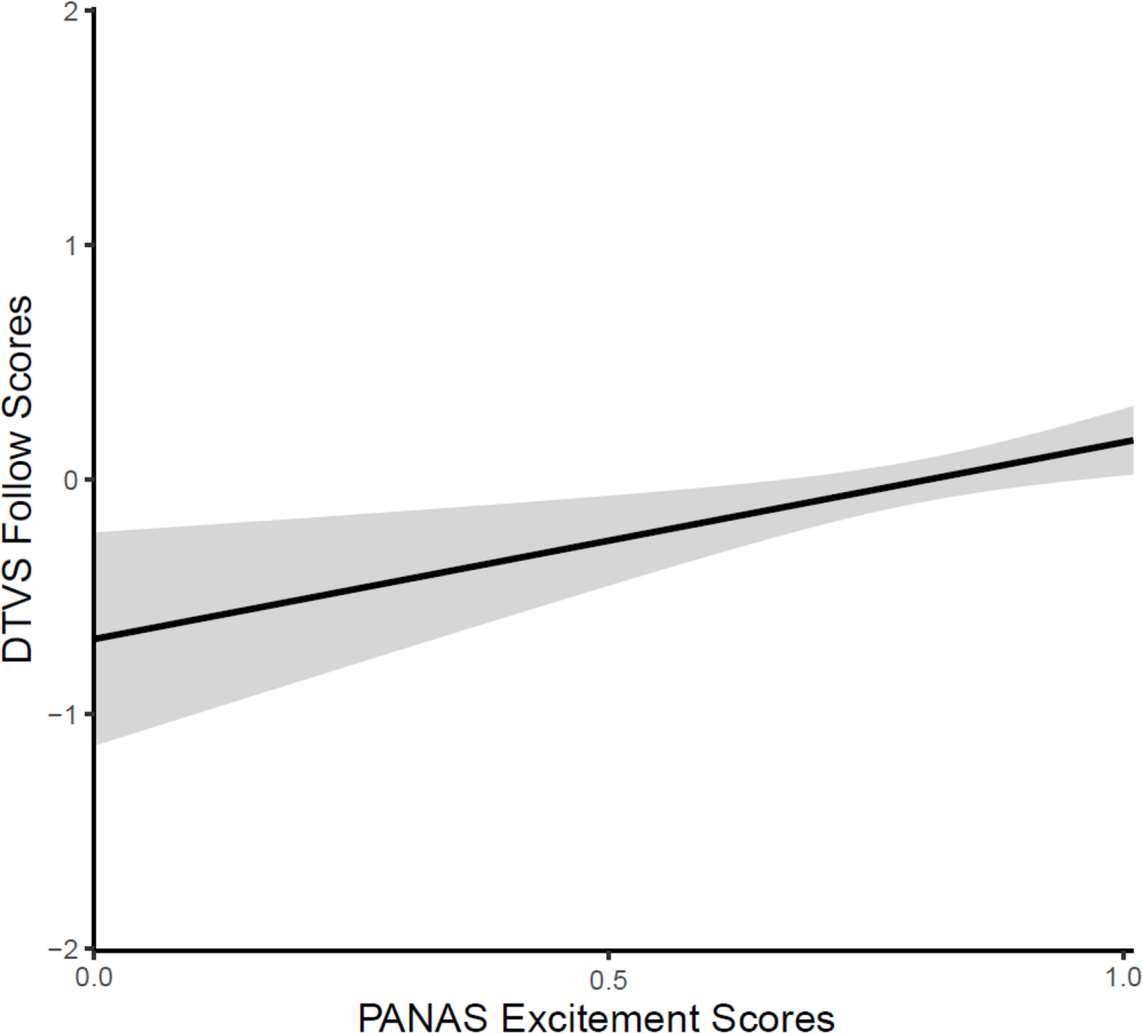
The effect of PANAS Excitement scores on DTVS Follow scores. Confidence bands represent a 95% confidence interval.

There were no significant effects or interactions in the prior experience model. Attend length did not have a significant effect on DTVS Follow scores, indicating that follow-related behaviors were not influenced by the amount of time that dogs attended to the television. Given that follow-related behaviors represent a different type of behavioral attendance than more passive behaviors, this was not considered a reflection of the validity of this component item. Breed grouping and neuter status did not significantly impact DTVS Follow scores, and therefore were excluded from the individual characteristics model. There were no significant effects of age or sex in the individual characteristics model. PANAS Excitement scores had a significant effect on Follow scores (*z* = 3.02, *p* = 0.002), such that dogs who had higher PANAS Excitement scores had higher Follow scores on average (see Fig. 2). None of the other PANAS or DIAS subscale items were significant.

#### DTVS non-animal

**Fig. 3:**
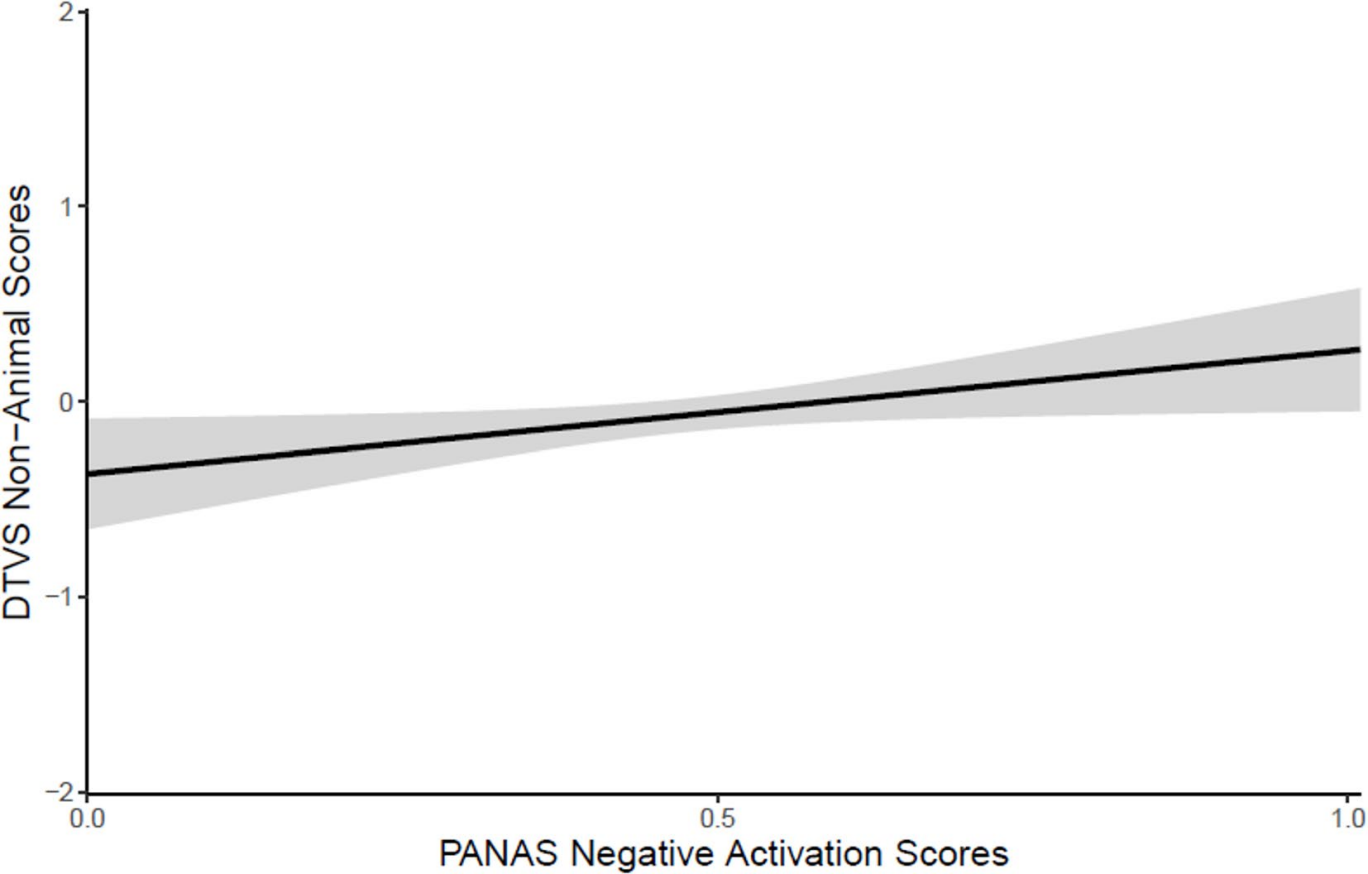
The effect of PANAS Negative Activation scores on DTVS Non-Animal scores. Confidence bands represent a 95% confidence interval.

There were no significant effects or interactions in the prior experience model. Attend length had a significant impact on Non-Animal scores, such that dogs who attended to television for longer had higher Non-Animal scores on average (*z* = 2.11, *p* = 0.035). Breed grouping and neuter status did not significantly impact DTVS Non-Animal scores, and therefore were excluded from the individual characteristics model. Neither age nor sex was significant in the individual characteristics model. There was a significant effect of PANAS Negative Activation scores on Non-Animal scores (*z* = 2.18, *p* = 0.029), such that dogs with higher PANAS Negative Activation scores had higher Non-Animal scores on average (see Fig. 3). None of the other PANAS or DIAS subscale items were significant.

## Discussion

The current study aimed to evaluate which characteristics of television stimuli are most influential on dogs’ engagement with television. In addition, this study aimed to evaluate what individual factors are most likely to impact these viewing behaviors. Results indicated that dogs interact with televisions, specifically differentiating between non-human animal stimuli and non-animal stimuli. Furthermore, there were differences in the types of behavior exhibited, indicating television interactions at the level of observational behaviors and interactions at the level of anticipatory following behaviors.

As a result of this study, a novel Dog Television Viewing Scale (DTVS) was formed, that addresses dogs’ behavior towards television across multiple sensory domains and stimulus categories. The PCA found that variation in responses was best explained by three component scores, two of which concerned the type of stimuli (i.e., animal, non-animal) and one of which concerned the type of behavior (i.e., following). Notably, scores did not load onto the visual and auditory components of the survey, suggesting that the type of stimuli presented is more influential than the sensory domain through which the stimulus is presented. This result is in line with previous findings in which behavioral differences in engagement were influenced by the type of stimuli presented through the television^1^. Furthermore, the loading of items onto a component that concerned following behavior is supportive of recent findings of anticipatory looking in dogs^11^, though rates with which dogs displayed anticipatory-type behavior in this sample were evenly distributed (i.e., not all dogs displayed high levels of anticipatory follow behaviors). In addition, television engagement was not found to be shaped by prior experiences (e.g., amount of television exposure) or by many individual characteristics of the dog (e.g., sex, breed group, age). However, some measures of temperament did impact dog engagement, suggesting that factors such as temperamental excitement levels and negative arousal levels should be considered when addressing rather positive or negative dog reactions to televisions. The results are supportive of the hypothesis that negative activation would result in increased television responsivity, indicating that negative arousal is linked to greater engagement with television stimuli— specifically television stimuli that represent inanimate objects or humans. Sex, age, and impulsivity measures did not significantly affect DTVS component scores, suggesting that neither increased potential for television interactions with age nor the dog-specific factors of sex and impulsivity levels are predictive of television viewing behaviors. Breed group did not significantly affect DTVS component scores, which may have resulted from the use of broader breed categories (e.g., hound versus herding groups) instead of specific breeds (e.g., sight hounds could be expected to perform different than scent hounds). However, we were unable to collect a sufficient number of responses at the breed-specific level to examine breed in this way.

While this study provides a measure for examining television viewing in dogs, there are some limitations. First, there was a self-selection of owners for those with dogs who do engage with television stimuli. While recruitment specifically encouraged owners to participate regardless of their dog’s viewing behaviors (or lack thereof), the vast majority of responses still consisted of dogs who regularly engage with the television. Therefore, questions concerning what factors may make a dog more or less likely to respond to television could only be partially answered, with important effects potentially masked due to the biased sample. Furthermore, these results cannot inform towards the rate at which dogs in the population watch television, only the factors that influence viewing within a sample of dogs that do. In addition, given the survey nature of the study, accurate owner interpretation of a dog’s behavior could not be guaranteed. While examples of behavior were provided and owners most likely are familiar with the tendencies of their dogs, accurate responding could not be verified. This limitation also prohibited the examination of behaviors based upon valence (i.e., positive or negative) or perceived intention. As such, this survey could be viewed as a jumping off point, with future studies examining these behaviors in laboratory or citizen science settings where behavior coding could be standardized and greater consistency over the types of programming presented could be performed. Additionally, questions concerning the size and type (e.g., LED, OLED) of television were collected, but the study was unable to analyze the effect of television type on viewing behaviors due to the representation of multiple television types within single households. In the future, examination of the impact of these factors on viewing habits would be recommended, as they may impact the quality of the stimuli and thus the perceptual experience of the dog^6^. Studies have been conducted demonstrating that dogs can accurately perceive video-projected images^5^, but that does not mean that these images are optimized for canine vision. Similarly, information concerning the types of television programming the owner plays for the dog and the daily routines of the dog (e.g., enrichment activities, exercise, training) was either not collected or unable to be analyzed. The prevalence of certain types of television programming in a home could influence owners’ interpretations of their dog’s behavior, even though the scale was purposefully designed to frame questions in terms of the frequency of behaviors rather than presentations of a certain type of object. Information concerning the enrichment activities, exercise prevalence, and training experiences of a dog could also have been beneficial, as it could be supposed that the daily routine of a dog may influence the likelihood of them attending to a television (e.g., a dog with frequent exercise and enrichment opportunities may be resting instead of attending to a television when it is on). It is recommended that future studies examine the influence of these factors on television viewing behaviors.

Despite the limitations of this study, this research provides valuable information for future exploration of television viewing in dogs, specifically as it concerns dog welfare needs. Studies have aimed to examine how television access can impact the welfare of dogs in shelter environments. While these studies have noted a lack of engagement^12^ or no decrease in cortisol levels^13^ as a result of television exposure, greater understanding of the factors influencing viewing behavior (e.g., a dog’s temperament, the type of television programming) could inform towards the ideal implementation of these welfare interventions. Additionally, this study found temperamental differences in dogs’ engagement with varying types of stimuli. Dogs who were more excitable were more likely to exhibit behaviors suggesting an expectation that the television stimulus exists in the 3D environment (i.e., follow behaviors). Furthermore, dogs who displayed more fearful tendencies were more likely to respond to the non-animal stimuli (e.g., car, doorbell). In situations where problem behaviors are being exhibited towards television media, these temperament-level differences could inform towards the best training practices to address such problems.

Lastly, this research informs towards the use of 2D stimuli in research tasks. The question of picture-object recognition (i.e., does a dog understand the referential nature of a 2D image) has long been debated in comparative cognition^14^. 2D visual stimuli are commonly used in animal tasks^14^ and in dog neuroimaging^15^, making dog use of television relevant to the discussion of these concepts. While this study did not directly examine picture-object recognition in dogs, the loading of the PCA components onto different natural stimulus categories suggests an accurate representation of objects even in the 2D modality.

These findings provide a foundation for future studies examining television viewing in dogs. We recommend that future studies use these findings to guide researchers towards what stimulus categories may be most beneficial in experimental studies. This study also provides important information regarding what individual factors may influence a dog’s viewing behaviors (e.g., negative activation). However, as previously stated, examination of these behaviors in an experimental setting in which image quality, types of programming, and standardized behavior coding can be conducted is warranted. Nevertheless, further evaluation of these behaviors could result in appropriate application of television interventions in shelters based upon individual dog temperament; maximization of the utility of television programming as an enrichment tool for companion dogs; and screening of television programming that could be a potential stressor for companion dogs.

## Conclusion

In this study, a novel Dog Television Viewing Scale (DTVS) was designed, allowing a standardized examination of the factors influencing dog television engagement behaviors. The results suggested that dogs respond to television stimuli based upon both categories of stimuli (i.e., animal, non-animal) and upon types of behavior (i.e., behavior that suggests attendance to the stimuli versus behavior that suggests some expectation of the movements of the stimuli). In addition, individual differences in temperament impacted the types of stimuli that dogs engaged with. This study informs towards the use of television as a welfare intervention, as well as dogs’ perceptual evaluations of artificial visual and auditory stimuli. Overall, this study indicates that companion dogs experience a meaningful, object-filled world when they view television.

## Electronic supplementary material

Below is the link to the electronic supplementary material.

Supplementary Material 1

## Data Availability

Access to data files is available upon request from the corresponding author, Lane Montgomery, via email (lim0004@auburn.edu).
