# Supplementary material for: Characterizing TV viewing habits in companion dogs
Source: Sci Rep. 2025 Jul 17;15:20274. doi: 10.1038/s41598-025-06580-y (PMC12271434; doi:10.1038/s41598-025-06580-y)
Supplement: Supplementary file 1 — Supplementary Material 1 [file 41598_2025_6580_MOESM1_ESM.pdf]

Supplementary Information

Title: Characterizing TV Viewing Habits in Companion Dogs

Journal: *Scientific Reports*

Authors: Lane I. Montgomery*, Sarah Krichbaum, and Jeffrey S. Katz

*Department of Psychological Sciences, Auburn University, lim0004@auburn.edu

Table S1. Survey questions concerning the dog's general TV viewing habits

Question	Response Options
Does your dog or has your dog watched TV?	○ Yes<br>○ No
How many hours, on average, is your dog exposed to TV each week?	[Free response]
Do you play TV for your dog when you are not home or are working?	○ Yes<br>○ No<br>○ Sometimes
If so, how old was your dog when you started playing TV for them?	[Free response]
How often does your dog engage with the TV (e.g., paw at, bark at, eyes follow)?	○ Never<br>○ Rarely<br>○ Sometimes<br>○ Often<br>○ Always<br>○ Not Observed
On average, how long will your dog attend to the TV (i.e., show attention to the TV without looking away or engaging with other stimuli)?	[Free response]
Does your dog attend to particular types of shows or movies?	○ Yes<br>○ No
If so, what particular types of TV does your dog attend to?	[Free response]
Have you tried to teach your dog to watch TV (e.g., point at the TV, play dog specific programs)?	○ Yes<br>○ No
What type of screen does your TV have? (If you have multiple TVs, you may select multiple screen types if necessary)	□ OLED<br>□ LED<br>□ LCD<br>□ Plasma<br>□ CRT<br>□ 4K<br>□ Uncertain<br>□ Other
How large is your TV? (If you have multiple TVs, you may select multiple TV sizes if necessary)	□ 39 inches or smaller (small)<br>□ 40 to 60 inches (medium)<br>□ Greater than 60 inches (large)<br>□ Uncertain
If you have the exact make, model, and dimensions of your TV, please fill them in below. (If you have multiple TVs, you may include this information for them all)	[Free response]

Additional questions concerning television viewing habits. Circle response options represent items for which a single response choice was allowed, while square response options represent items for which multiple response choices were allowed.

Table S2. Breakdown of responses for general TV viewing questions within the final sample

Question	Response Options	Percentage () /number [] of responses
Do you play TV for your dog when you are not home or are working?	○ Yes<br>○ No<br>○ Sometimes	○ Yes **(25%)**<br>○ No **(54%)**<br>○ Sometimes **(21%)**
How often does your dog engage with the TV (e.g., paw at, bark at, eyes follow)?	○ Never<br>○ Rarely<br>○ Sometimes<br>○ Often<br>○ Always<br>○ Not Observed	○ Never **(0%)**<br>○ Rarely **(.8%)**<br>○ Sometimes **(5%)**<br>○ Often **(24%)**<br>○ Always **(54%)**<br>○ Not Observed **(16%)**
Does your dog attend to particular types of shows or movies?	○ Yes<br>○ No	○ Yes **(87%)**<br>○ No **(13%)**
Have you tried to teach your dog to watch TV (e.g., point at the TV, play dog specific programs)?	○ Yes<br>○ No	○ Yes **(15%)**<br>○ No **(85%)**
What type of screen does your TV have? (If you have multiple TVs, you may select multiple screen types if necessary)	□ OLED<br>□ LED<br>□ LCD<br>□ Plasma<br>□ CRT<br>□ 4K<br>□ Uncertain<br>□ Other	□ OLED **[60]**<br>□ LED **[230]**<br>□ LCD **[58]**<br>□ Plasma **[21]**<br>□ CRT **[0]**<br>□ 4K **[21]**<br>□ Uncertain **[109]**<br>□ Other **[20]**
How large is your TV? (If you have multiple TVs, you may select multiple TV sizes if necessary)	□ 39 inches or smaller (small)<br>□ 40 to 60 inches (medium)<br>□ Greater than 60 inches (large)<br>□ Uncertain	□ 39 inches or smaller (small) **[66]**<br>□ 40 to 60 inches (medium) **[297]**<br>□ Greater than 60 inches (large) **[163]**<br>□ Uncertain **[2]**

Additional questions concerning dog television habits with the number/percentage of responses for each answer option included. Reported numbers in parentheses represent percentages, while reported numbers in brackets represent counted number of responses.

Table S3. Number of responses for DTVS items within the final sample

For the following visual TV items, please state how often your dog follows those types of objects off-screen. Following includes (but is not limited to) behaviors such as looking behind the TV for an object and walking alongside an object as it leaves the screen.

	Never	Rarely	Sometimes	Often	Always	Not Observed
Dogs	142	49	83	90	89	1
Non-dog household pets (e.g., cats)	172	57	92	86	47	0
Non-household animals (e.g., horses, birds)	161	60	84	105	44	0
Humans	246	89	85	28	5	1
Inanimate objects (e.g., cars)	286	87	64	13	3	1

For the following visual TV items, please state how often your dog responds to those types of objects. Responding includes (but is not limited to) behaviors such as pawing at the TV, tail wagging, ear movements, and growling.

	Never	Rarely	Sometimes	Often	Always	Not Observed
Dogs	0	13	33	80	144	183
Non-dog household pets (e.g., cats)	1	45	65	131	126	85
Non-household animals (e.g., horses, birds)	1	45	62	115	143	87
Humans	0	139	152	120	37	5
Inanimate objects (e.g., cars)	236	111	81	21	4	2

For the following auditory TV items, please state how often your dog responds to those types of noises. Responding includes (but is not limited to) behaviors such as tail wagging, ear movements, barking, and whining.

	Never	Rarely	Sometimes	Often	Always	Not Observed
Dog noises (e.g., barking, howling)	11	18	71	148	206	0
Non-dog household animal noises (e.g., meowing)	45	83	147	107	72	1
Non-household animal noises (e.g., neighing, mooing)	57	91	161	91	51	4
Human noises (e.g., talking, yelling)	151	140	112	48	4	0
Inanimate object noises (e.g., car horn, doorbell)	93	103	134	87	38	0
Weather noises (e.g., thunder, rain)	207	127	84	24	6	6

The DTVS with the number of responses for each answer option included. Numbers represent a numerical count of responses for each answer choice.

Table S4. PCA component loadings

**Following**	
Dogs	Component 2
Non-dog household pets	Component 2
Non-household animals	Component 2
Humans	Component 2
Inanimate objects	Component 2; Component 3
**Visual**	
Dogs	Component 1
Non-dog household pets	Component 1
Non-household animals	Component 1
Humans	Component 3
Inanimate objects	Component 3
**Auditory**	
Dog noises	Component 1
Non-dog household animal noises	Component 1
Non-household animal noises	Component 1
Human noises	Component 3
Inanimate object noises	Component 3
Weather noises	Component 3

The items of the DTVS with the corresponding component loadings listed to the right.

Figure S1. Distribution of scores for PCA components

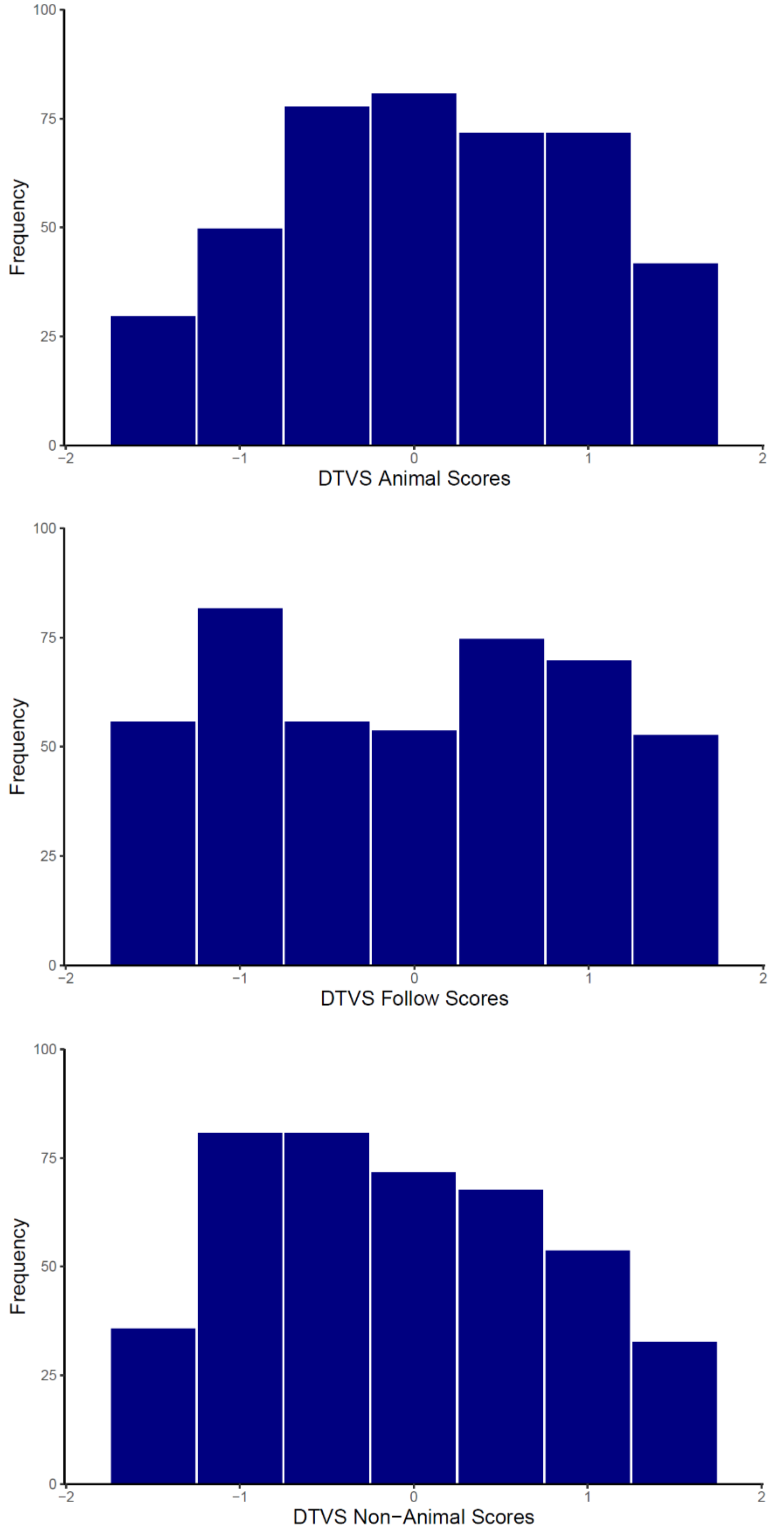

The distribution of scores within the sample for the DTVS PCA components.
